# A Deep Insight into the Sialome of *Rhodnius neglectus*, a Vector of Chagas Disease

**DOI:** 10.1371/journal.pntd.0004581

**Published:** 2016-04-29

**Authors:** Paula Beatriz Santiago, Teresa C. F. Assumpção, Carla Nunes de Araújo, Izabela Marques Dourado Bastos, David Neves, Ionizete Garcia da Silva, Sébastien Charneau, Rayner Myr L. Queiroz, Tainá Raiol, João Victor de Araújo Oliveira, Marcelo Valle de Sousa, Eric Calvo, José M. C. Ribeiro, Jaime M. Santana

**Affiliations:** 1 Department of Cell Biology, The University of Brasília, Brasília, Brazil; 2 Vector Biology Section, Laboratory of Malaria and Vector Research, National Institute of Allergy and Infectious Disease, Rockville, Maryland, United States of America; 3 Ceilândia Faculty, The University of Brasília, Brasília, Brazil; 4 Department of Parasitology, The University of Goiás, Jataí, Brazil; 5 Instituto Leônidas e Maria Deane - Fiocruz Amazônia, Manaus, Brazil; 6 Department of Computer Science, The University of Brasília, Brasília, Brazil; Fundaçao Oswaldo Cruz, BRAZIL

## Abstract

**Background:**

Triatomines are hematophagous insects that act as vectors of Chagas disease. *Rhodnius neglectus* is one of these kissing bugs found, contributing to the transmission of this American trypanosomiasis. The saliva of hematophagous arthropods contains bioactive molecules responsible for counteracting host haemostatic, inflammatory, and immune responses.

**Methods/Principal Findings:**

Next generation sequencing and mass spectrometry-based protein identification were performed to investigate the content of triatomine *R*. *neglectus* saliva. We deposited 4,230 coding DNA sequences (CDS) in GenBank. A set of 636 CDS of proteins of putative secretory nature was extracted from the assembled reads, 73 of them confirmed by proteomic analysis. The sialome of *R*. *neglectus* was characterized and serine protease transcripts detected. The presence of ubiquitous protein families was revealed, including lipocalins, serine protease inhibitors, and antigen-5. Metalloproteases, disintegrins, and odorant binding protein families were less abundant.

**Conclusions/Significance:**

The data presented improve our understanding of hematophagous arthropod sialomes, and aid in understanding hematophagy and the complex interplay among vectors and their vertebrate hosts.

## Author Summary

Chagas disease is caused by the *Trypanosoma cruzi* protozoan, which is transmitted to vertebrates through the feces of infected triatomines during blood sucking. The vascular injury caused by the bite triggers mechanisms capable of preventing the association with hosts, such as immune response, inflammation and haemostasis. However, hematophagous insects are able to counteract these defenses through a complex repertoire of salivary molecules that have specific targets in the host. Our results show that *R*. *neglectus* salivary glands express different protein gene families, possessing multi-functional features directly related to different anti-haemostatic activities. For instance, lipocalins are proteins possessing anti-coagulant and vasodilator functions. Saliva contents have evolved to adapt to blood-feeding habit, ensuring the maintenance of blood flow, the success of the meal, and transmission of diseases.

## Introduction

Blood-sucking triatomines (Hemiptera: Reduviidae) feed exclusively on blood in all life stages. They obtain their blood meal from venules or arterioles of their vertebrate hosts. The steps during feeding include piercing of the host skin, followed by a probing period, and finally engorgement [1]. In support of this habit, these arthropods have evolved effective mechanisms to counteract host responses, such as haemostasis, inflammation and immunological reactions. While biting, their salivary glands (SG) release potent pharmacological substances, including vasodilator, anti-inflammatory, antiplatelet, anticlotting and immunomodulatory molecules, to enable the arthropod to obtain a successful blood-meal [2, 3]. These bioactive salivary components represent a promising source of molecules with therapeutic potential for treating circulatory disorders [4, 5].

In the 1990s, multinational control programs against Chagas disease led to a significant reduction of acute cases in many endemic regions of Latin America, mainly through a reduction of domestic vectors [6]. However, factors such as the wide geographical distribution of triatomine species and the availability of different infection reservoirs remain multifactorial obstacles in the control of the disease. Nowadays, there is constant concern regarding the sporadically or progressive (re)invasion and (re)colonization of human dwellings by wild secondary vectors [7, 8]. *Rhodnius neglectus* is found in the Brazilian Savanna (*Cerrado*) in association with different wild palms, playing an important role in the sylvatic maintenance of *T*. *cruzi* and *Trypanosoma rangeli* [9–11]. In nature, *R*. *neglectus* feeds mainly on birds and much less on rodents, and rarely on opossum [12]. This species is able to act as a secondary vector, being observed in both intra and peridomestic environments in five Brazilian states [13–17], a possible result of deforestation and wild ecotope invasion. These anthropogenic environmental changes favor vector dispersion, bridging sylvatic/domestic cycles of the disease.

Sialome studies (from the Greek sialo = saliva) have been developed for many species of bloodsucking insects, which are frequently vectors of human and animal diseases. Sanger automated sequencing technology has been used to investigate the salivary transcriptome for almost two decades. However, Next Generation Sequencing (NGS) is capable of providing much more sequence data in a single run, with a higher resolution than that from the Sanger technique, allowing for deeper analysis of the transcripts. One important application of NGS is RNA sequencing (RNA-seq), used to describe transcriptomes of cells and tissues. Deep sequencing increases the possibilities of finding new biological molecules in the saliva of bloodsucking insects, offering a new array of substances to be further investigated and functionally characterized.

The aim of this report is to catalog the transcripts of *R*. *neglectus* SGs with probable function in hematophagy using RNAseq and mass spectrometry. This strategy was used to describe the bioactive molecules in triatomine saliva and improve our understanding on the dynamics of the blood-feeding process, vector-host interaction and disease transmission. The data is available at the National Center for Biotechnology Information (NCBI) and can be used in different scientific research projects.

## Methods

### Insects and Transcriptome Salivary Gland Preparation

*R*. *neglectus* triatomines originating from insects collected in 1982 at Itambaracá, in Paraná State, Brazil, were reared in the insectarium at the University of Brasília (Brazil). They were kept at 27±1°C, a relative humidity of 70–75%, under a 12 h/12 h light/dark cycle. The blood source of these insects was *Gallus gallus domesticus*. The SGs of 5^th^ instar nymphs and adults were dissected at 5, 12, and 24 days post blood meal in cold Trizol reagent (Invitrogen, Carlsbad, CA, USA). A pool of thirty SG pairs was stored at -80°C prior to RNA extraction.

### Salivary Gland RNA Isolation, Library Preparation and Sequencing

Total RNA was extracted following the Trizol manufacturer’s instructions. RNA integrity and concentration were checked by lab-on-chip analysis using an Agilent 2100 Bioanalyzer (Agilent Technologies, USA). A RNA sample was sent to the Federal District High-Performance Genome Center (DF, Brazil) for Illumina cDNA library construction and next generation sequencing. A Library was prepared with standard protocols using TruSeq RNA kit, v2 (Illumina, San Diego, CA). To generate paired-end reads of 300 nucleotides in length, the sequencing of cDNA libraries was performed on an Illumina MiSeq sequencer (Illumina, USA). One lane of the MiSeq machine was used for sequencing this and another library, distinguished by bar coding. The RNA-seq sequencing generated a total of 12,049,305 reads. The nominal length of the sequences was 301 nt. Following trimming of low quality bases (quality 20 or lower), the average length was 248.07, the median was 301 and L50 was 296 nt. Sequences smaller than 25 nt or with average quality < 20 were rejected.

### Bioinformatic Analysis

Bioinformatic analyses were conducted as previously described [18]. As there was no reference genome to map, the strategy was to perform a *de novo* assembly with Abyss [19] and Soapdenovo Trans [20] assemblers using different kmer (k) values (from 20 to 90). The resulting assemblies were joined by an iterative BLAST and cap3 assembler [21]. Sequence contamination between bar-coded libraries were identified and removed when their sequence identities were over 98%. Coding sequences (CDS) were extracted based on the existence of a signal peptide and on similarities to other known proteins [22]. Coding and protein sequences were mapped into a hyperlinked Excel spreadsheet. Reads were mapped into contigs using blastn [23] with a word size of 25, masking homonucleotide decamers and allowing mapping to up to five different CDS if the BLAST results had the same scores. Mapping of the reads was also included in the Excel spreadsheet. CDS were automatically annotated a program written by JMCR that searched a vocabulary of nearly 250 words for matches various databases, including Swissprot, Gene Ontology, KOG, PFAM, and SMART, and a subset of the non-redundant protein database containing proteins from vertebrates (NCBI). Further manual annotation was done as required. Alignment analysis were done with Bioedit software [24] after sequence alignment performed using ClustalW [25]. Phylogenetic analysis and statistical neighbor-joining bootstrap tests of the phylogenies were done with Mega package [26]. The sequences used in alignments with *R*. *neglectus* CDS were obtained from the non-redundant protein database of the NCBI and are represented by six letters followed by the NCBI GI number. The letters derive from the first three letters of the genus and the first three letters of the species name.

### Data Availability

The raw reads were deposited at the Sequence Read Archive (SRA) in NCBI under bioproject PRJNA292130. A total of 4,230 coding sequences were deposited in DDBJ/EMBL/GenBank through the Transcriptome Shotgun Annotation portal under the accession GDKW00000000.

### LC-MS/MS Protein Identification

The SGs were dissected from 5^th^ instar nymphs and adults at 5, 12 and 24 days post blood meal and carefully punctured at 4°C. Following centrifugation (16.000 × g, 15 min, 4°C), the soluble protein fraction from fifteen pairs of SG homogenates was ethanol/acetone precipitated. Resuspended proteins were consecutively alkylated, reduced, digested by trypsin, and subjected to LC-MS/MS analysis as previously described [27]. Briefly, the tryptic peptides were loaded onto a 2 cm fused silica trap column (150 μm inner diameter) packed in-house with reverse phase capillary column ReproSil-Pur C18-AQ 5 μm resin (Dr. Maisch GmbH, Germany) and separated using a DIONEX 3000 nanoUPLC system coupled to an LTQ-Orbitrap Elite mass spectrometer (Thermo Scientific, Waltham, USA). MS1 spectra were recorded in the Orbitrap mass analyzer with 120,000 resolution. After ion fragmentation, MS/MS spectra of the 15 most intense ions were acquired. Raw files were generated and used for protein identification using Proteome Discoverer v.1.3 (Thermo Scientific, Waltham, USA) with in-house SequestHT algorithm for *R*. *neglectus* SG transcriptome and human keratins, BSA and porcin trypsin. The false discovery rate was less than 1%, with peptide rank of 1 and at least 2 peptides per protein.

## Results and Discussion

### General Description of the Sialome of *R*. *neglectus*

The assembly of *R*. *neglectus* SG transcriptome enabled the extraction of 5,705 CDS. These CDS mapped a total of over 11 million reads. Following automated and manual annotation, the CDS were classified into putative secreted, housekeeping, unknown, transposable element, and viral product. The CDS of the housekeeping class comprised the largest class (Table 1). They were further characterized into 24 subclasses, according to their possible function, summarized in Table 2.

**Table 1:** Classification and abundance of coding sequences extracted from the salivary gland transcriptome of *R*. *neglectus*.

Class	No. of CDS	% Total	No. of reads	% Total
Secreted	636	11.15	2,978,414	25.44
Housekeeping	4,739	83.07	8,320,391	71.07
Unknown product	242	4.24	367,035	3.13
Transposable element	86	1.51	41,406	0.36
Viral product	2	0.03	208	0.00
**Total**	**5,705**	**100**	**11,707,454**	**100**

**Table 2:** Classification and abundance of coding sequences of putative housekeeping function extracted from the sialotranscriptome of *R*. *neglectus*.

Subclass	No. of CDS	No. of reads	% Total
Unknown conserved	805	1,219,305	14.65
Protein export	271	1,151,192	13.84
Protein synthesis machinery	287	779,314	9.37
Signal transduction	604	725,961	8.73
Protein modification	153	595,681	7.16
Transcription machinery	468	566,268	6.81
Lipid metabolism	217	477,020	5.73
Nucleotide metabolism	87	426,894	5.13
Cytoskeletal protein	190	297,617	3.58
Transporter and Channel	272	293,911	3.53
Protein modification, protease	91	244,360	2.94
Carbohydrate metabolism	150	230,497	2.77
Immunity	78	177,405	2.13
Extracellular matrix	92	175,813	2.11
proteasome	191	157,781	1.90
Energy metabolism	166	143,638	1.73
Amino acid metabolism	79	127,157	1.53
Nuclear Export	26	120,662	1.45
Nuclear Regulation	218	116,946	1.41
Transcription factor	100	99,589	1.20
Detoxification	93	77,717	0.93
Storage	15	56,888	0.68
Intermediary metabolism	57	30,227	0.36
Signal Transduction, apoptosis	28	28,548	0.34
**Total**	**4739**	**8,320,391**	**100**

### Putative Secreted Proteins

The secreted class was organized in subclasses that include previously known gene families present in hematophagous saliva, such as lipocalin, nitrophorin, antigen-5, as well as gene families not commonly reported in triatomine saliva, such as serine protease and disintegrin (Table 3). The following section describes the putative secreted proteins present in *R*. *neglectus* sialome, highlighting the remarkable finding of serine proteases in this group.

**Table 3:** Classification and abundance of coding sequences of putative secretory function extracted from the sialotranscriptome of *R*. *neglectus*.

Subclass	No. of CDS	No. of reads	% Total
Hypothetical secreted protein	198	976,296	32.78
Serine protease	33	820,619	27.55
Conserved secreted protein	89	529,615	17.78
Lipocalin—Triabin	120	471,408	15.83
Disintegrin	2	62,933	2.11
Others	64	52,690	1.77
Lipocalin—Nitrophorin	31	13,737	0.46
Mucin related	11	11,410	0.38
Antigen-5/SCP	8	8,718	0.29
Lipid metabolism	16	6,028	0.20
Major royal jelly protein	2	5,019	0.17
Juvenile hormone related	8	4,328	0.15
Protease inhibitor	14	3,164	0.11
Immunity related	4	2,829	0.09
Insect pheromone-binding	6	2,769	0.09
Protease inhibitor Kazal—type	7	1,993	0.07
OBP	11	1,837	0.06
Toxin	1	1,542	0.05
Nucleotid metabolism	6	735	0.02
5’ nucleotidase	3	477	0.02
Hemolysin-like	1	242	0.01
Metalloprotease	1	35	0.00
**Total**	**636**	**2,978,424**	**100**

### Lipocalins

Lipocalins comprised one of the most abundant groups of transcripts, with 16.29% of putatively secreted reads. These include a large group of extracellular proteins that usually bind to small hydrophobic molecules, cell surface receptors or other proteins. The members of this family have little similarity in peptide sequence, however share a conserved three-dimensional structure, comprised of a single eight-stranded antiparallel β-barrel [28]. In blood-sucking insect and tick saliva the lipocalins are abundantly expressed, but not in Diptera or fleas. In ticks, their function is associated with binding to histamine and serotonin [29]. Triabin and nitrophorin, the two major groups found here, are discussed below.

#### Lipocalins of the triabin family

**Fig 1:**
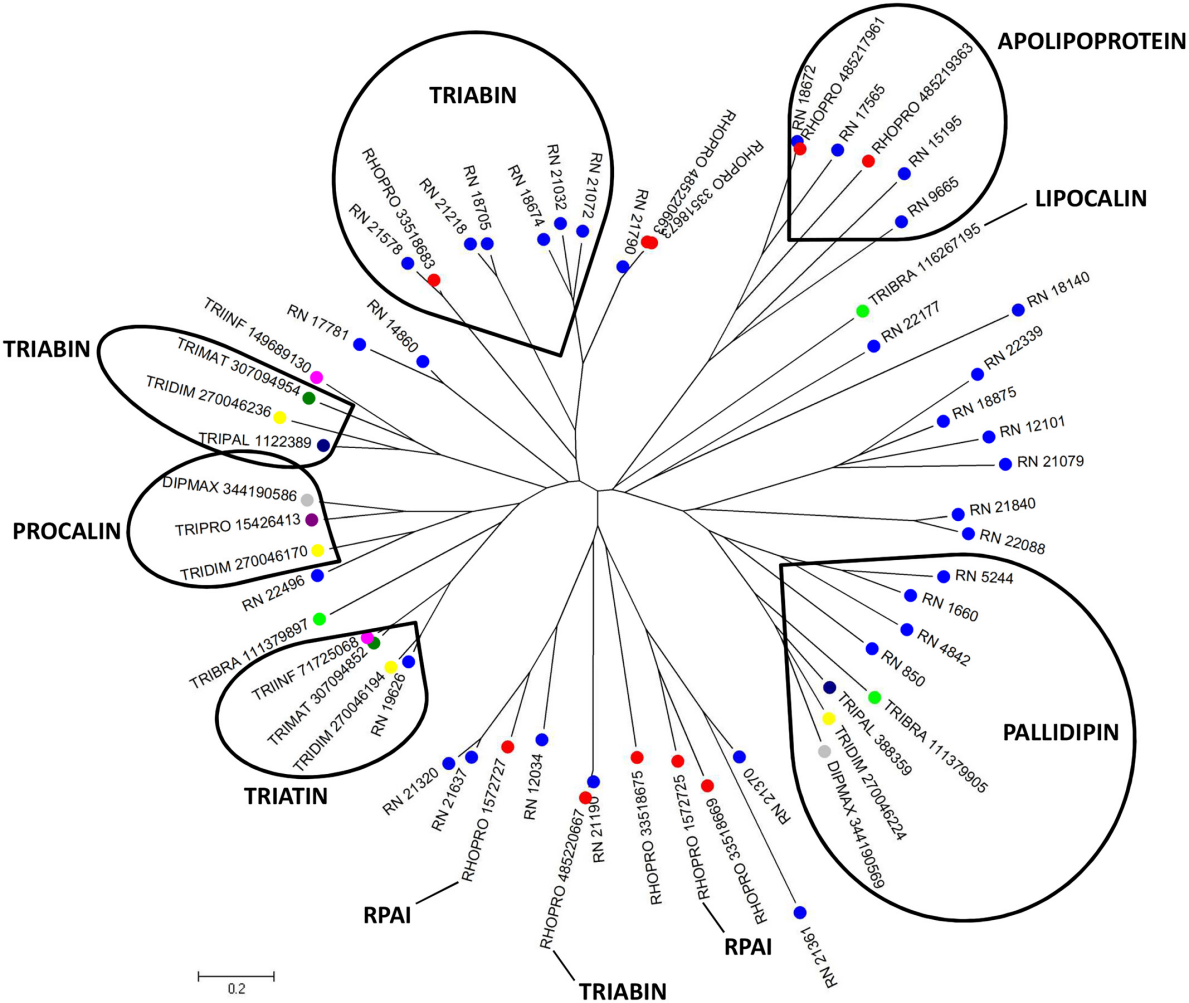
Phylogram of lipocalin containing triabin domain from *R*. *neglectus* SG transcriptome. Phylogenetic tree derived from the alignment of *R*. *neglectus* CDS and other triatomine lipocalin sequences as described in Methods section. The bar at the bottom represents 20% amino acid substitution. The colored circles indicate each species whose sequences were used: blue, *R*. *neglectus* sequences from SG transcriptome; red, *R*. *prolixus*; yellow, *Triatoma dimidiata*; green, *Triatoma brasiliensis*; dark green, *Triatoma matogrossensis*; dark blue, *T*. *pallidipennis*; purple, *Triatoma protacta*; magenta, *Triatoma infestans*; gray, *Dipetalogaster maxima*.

First isolated from the saliva of the *Triatoma pallidipennis* kissing bug [30], triabin is a lipocalin-like thrombin inhibitor, which inhibits thrombin-induced platelet aggregation, and prolongs thrombin clotting time through the formation of a noncovalent complex with thrombin at a 1:1 molar ratio. Previous analysis revealed that triabin is a compact one-domain molecule essentially consisting of an eight-stranded β-barrel and inhibits thrombin exclusively via its fibrinogen-recognition exosite [31]. Thrombin is the ultimate serine protease formed during activation of the blood coagulation cascade, which catalyzes the polymerization of fibrinogen to fibrin, the solid fibrillar component of the blood clot, thereby being a fundamental promoter of blood clotting. Thus, the triabin-like lipocalins may function as thrombin inhibitors in *R*. *neglectus* saliva. The library analysis shows 120 different CDS from lipocalin family containing the triabin conserved domain, such as triabin, pallidipin, apolipoprotein, procalin and triatin. The alignment of these members with lipocalins already described in triatomines resulted in a phylogram containing different clades (Fig 1). In addition, it is possible to note two divergent clades containing only *R*. *neglectus* and *Rhodnius prolixus* sequences (RPAI and Apolipoprotein), which may represent additional gene members present in *Rhodnius* spp. The presence of different clades indicates the expansion of this gene family by gene duplication events, suggesting that, for *R*. *neglectus*, lipocalins exert a crucial role in success feeding.

#### Lipocalins of the nitrophorin family

**Fig 2:**
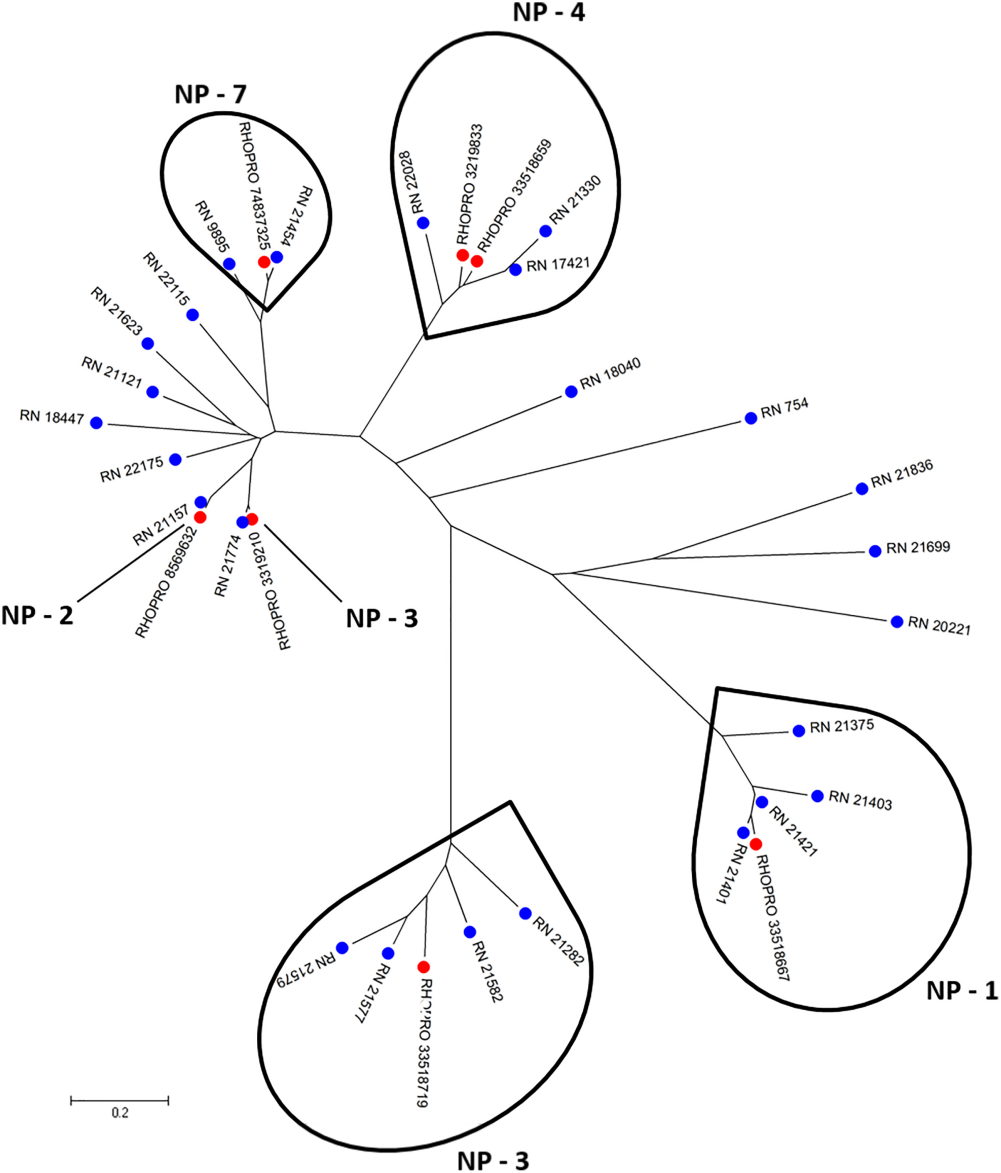
Phylogram of lipocalin containing nitrophorin domain from *R*. *neglectus* SG transcriptome. Phylogenetic tree derived from the alignment of *R*. *neglectus* CDS and *R*. *prolixus* nitrophorin sequences as described in Methods section. The bar at the bottom represents 20% amino acid substitution. The colored circles indicate each species whose sequences were used: blue, *R*. *neglectus* sequences from SG transcriptome and red, *R*. *prolixus* sequences from NCBI.

*Rhodnius* spp. show a characteristic red coloration in their saliva due to the presence of haemoproteins called nitrophorins (NPs). These molecules form a stable complex with nitric oxide (NO), which is sensitive to pH variation, being stabilized by low pH in the lumen of the SGs (pH ~5), and released at neutral pH in the host (pH ~7.5) [32]. The secretion of NO is an efficient way to counteract haemostasis, acting both as a potent vasodilator and as an antagonist of platelet activation. NPs 1–4 can additionally sequester histamine released by host mast cells, reducing inflammation and immune response [33, 34]. NP 2 inhibits clotting in a mechanism independent of NO or histamine binding, acting as a specific inhibitor of the intrinsic factor X-(FX)-activating complex [35]. As well as reversibly binding to NO or histamine, NP 7 also inhibits prothrombin activation by blocking phospholipid binding sites for the prothrombinase complex on the surfaces of vesicles and activated platelets through binding to phosphatidylserine [36]. The current sialotranscriptome identified 13,737 reads related to the diversity of NPs. The NPs of *R*. *neglectus* also appear to be a gene family that expanded during evolutionary processes, as inferred by the phylogenetic tree (Fig 2). Notice that there are several sequences homolog to NP1-4 and 7, NPs described in *R*. *prolixus* saliva.

The mean number of nitrophorins in salivary electrophoretic profiles varies among *Rhodnius* species, with *R*. *neglectus* showing the fewest. The high polymorphism of NPs may help in the identification of *Rhodnius* species [37]. The lower proportion of nitrophorin content in the saliva compared to those found in the saliva of other *Rhodnius* spp. might not, by itself, explain the reduced feeding performance of *R*. *neglectus* on mammals. For instance, although *R*. *neglectus* shows lower amounts of nitrophorins, it feeds more efficiently than *R*. *robustus* [37]. It is important to note that the exact contribution of each class of saliva molecules on the feeding process is unknown.

### Antigen-5 Family

The CAP superfamily members [Cysteine-Rich Secretory Proteins (CRISPS), Antigen 5 (Ag5), and Pathogenesis-Related 1 (Pr-1)] are found in a wide range of organisms, most often as secreted proteins [38]. Ag5, present in the venom of wasps and ants, are considered potent allergens to mammals [39, 40]. This superfamily can also block smooth muscle contraction when present in snake venom [41] and act in the defense response in plants [42]. They have been described in the saliva of some hematophagous, including mosquitoes [43, 44] and sand flies [45]. Among triatomines, Ag5 genes have been reported in the sialotranscriptomes of *R*. *prolixus* [46], *T*. *infestans* [47], *D*. *maxima* [48], *T*. *matogrossensis* [49] and *Triatoma rubida* [50]. Their functions in blood-feeder saliva remained unexplored for a long time, but a recent report revealed salivary Ag5 of *D*. *maxima* and *T*. *infestans* as Cu^+2^-dependent antioxidant enzymes that inhibit neutrophil oxidative burst and platelet aggregation induced by collagen [51].

**Fig 3:**
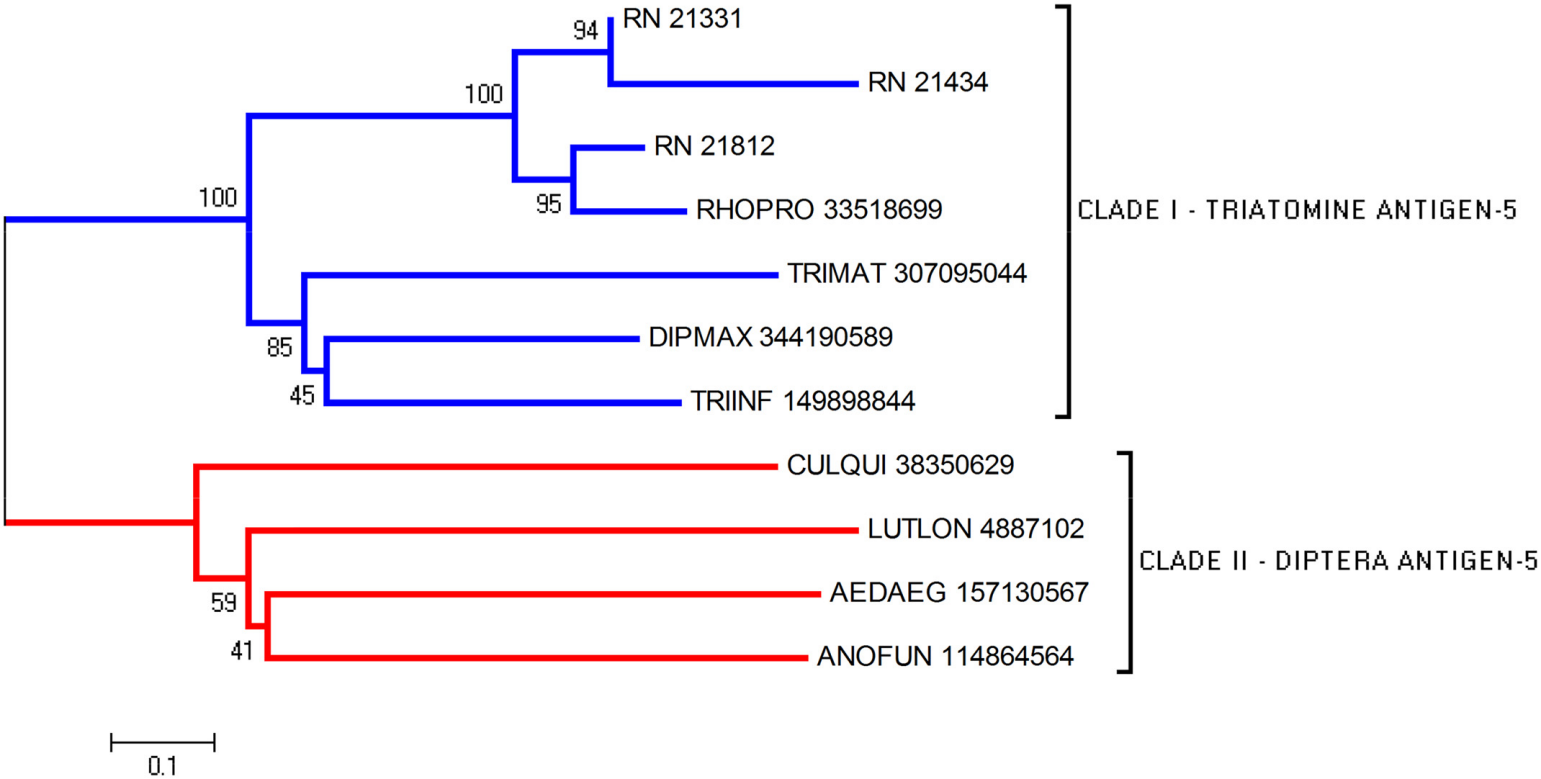
Phylogram of Antigen-5 proteins from *R*. *neglectus* SG transcriptome. Phylogenetic tree derived from the alignment of *R*. *neglectus* CDS and other insect antigen-5 sequences as described in Methods section. The bar represents 10% amino acid substitution.

The sialotranscriptome analysis revealed eight CDS related to the Ag5 family. The alignment of *R*. *neglectus* Ag5 with other triatomine Ag5 sequences showed some conserved motifs (S1 Fig). Phylogenetic analysis offers support for the formation of clades I and II comprising triatomine and Diptera sequences, respectively (Fig 3).

### Serine Protease Inhibitors

For blood-feeders, targeting components of the coagulation cascade is essential to attenuate the haemostatic response of their hosts. All enzymes participating in this cascade are serine proteases associated with complement activation [52, 53]. The *R*. *neglectus* sialotranscriptome exhibited a variety of transcripts coding for proteins with serine protease inhibitory function, comprising 14 CDS and 3,164 reads. Based on their Pfam signature, kazal, pacifastin and serpin families were extracted.

#### Kazal family

Kazal-type domain-containing proteins are serine protease inhibitors playing important functions in invertebrates, mainly having vasodilation, antimicrobial, and thrombin inhibition effects. These protease inhibitors are single or multidomain proteins that share a conserved sequence motif, a distinctive cysteine distribution pattern and highly similar three-dimensional structure [54]. Rhodniin is a kazal-type thrombin inhibitor isolated from *R*. *prolixus* [55, 56]. Dipetalogastin from *D*. *maxima* [57], infestin from *T*. *infestans* [58] and brasiliensin from *T*. *brasiliensis* [59] are thrombin inhibitors located in the intestines. From the horse fly *Hybomitra bimaculata* (Diptera, Tabanidae) SGs, a vasodilator named vasotab was identified as a member of Kazal-type protease inhibitor family acting through ion channel inhibition and vasodilation [60].

**Fig 4:**
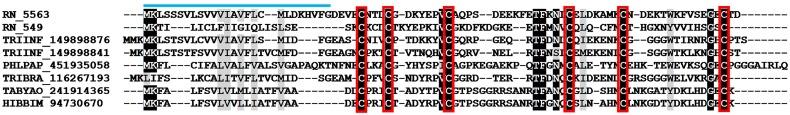
Kazal-type members from *R*. *neglectus* SG transcriptome. ClustalW alignment of Kazal-type domain-containing members from *R*. *neglectus* salivary transcriptome (RN_5563 and RN_549) and other insect kazal-type sequences, identified as described in Methods section. The alignment indicates conserved residues in black and similar residues in gray background, the six conserved cysteines (boxes) and the blue bar indicates the signal peptide indicative of secretion.

Seven CDS in *R*. *neglectus* sialotranscriptome possessed the typical sequence of nonclassical Kazal domains characterized by a shorter distance between the first and second cysteine residue, unlike the seven or eight spacer residues found in the classical configuration [55, 57]. The alignment showed a low degree of conserved amino acids, but confirmed the presence of the six cysteine residues responsible for the formation of disulfide bridges (Fig 4). The relative positions of cysteine residues were the same in the compared sequences.

Additionally, one contig was identified as dipetalogastin due to the cysteine residues distribution and the presence of the conserved motif CGXDXXTYXNXC, a distinguishing repeat of Kazal-type inhibitors [57]. This transcript is full length and possesses the signal peptide indicative of secretion. The alignment with other protein sequences with the same features revealed a high degree of conserved amino acids (S2 Fig).

**Fig 5:**
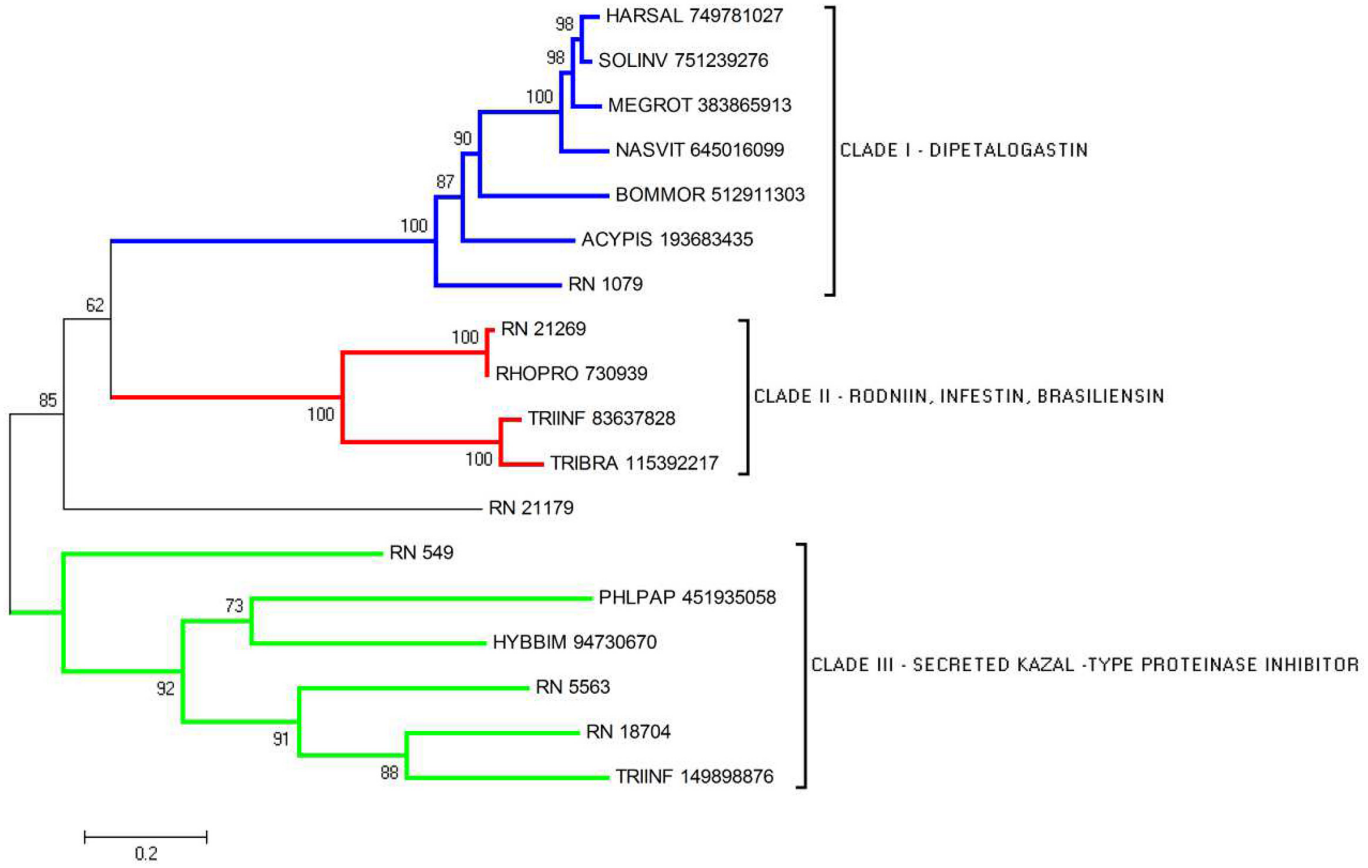
Phylogram of Serine Protease inhibitors from *R*. *neglectus* SG transcriptome. Phylogenetic tree derived from the alignment of *R*. *neglectus* CDS and other insect sequences as described in Methods section. The bar at the bottom represents 20% amino acid substitution.

The phylogram of serine protease inhibitor members clearly shows the formation of three clades, with a good bootstrap support, each one representing a different family of serine protease inhibitor discussed above (Fig 5). The CDS RN_21179 is notably distinct from the clades, suggesting the presence of a divergent gene. The different clades may represent sequences differentially expressed sharing the same function regarding haemostasis inhibition.

#### Pacifastin family

Pacifastin is a family of serine protease inhibitors, mostly multi-domain proteins, first isolated from the plasma of the crayfish *Pacifastacus leniusculus*. The protein is heterodimeric, comprising both a transferrin chain (heavy chain, PHC) and a protease inhibitor chain (light chain, PLC) [61, 62]. Insect pacifastins may have multiple functions, acting as regulators of a wide variety of serine peptidase-dependent processes such as immunity and reproduction [63]. In Hemiptera, two pacifastin-like protease inhibitors from *T*. *infestans* eggs were functionally characterized, suggesting a role in insect immune response [64]. Here, two CDS are related to pacifastin, RN_17301 and RN_20047, and their alignment with other members of the pacifastin family reveals four conserved domains, containing the cysteine-rich inhibitory pattern of PLC comprised of a triple-stranded antiparallel beta-sheet connected by three disulfide bridges (S3 Fig). This is the first time pacifastin members are identified in triatomine SGs, their function in this organ is still unknown but it might be related to insect immunity.

#### Serpin family

Serpins are a large family of structurally related proteins found across taxa, showing diverse activities not limited to inhibition of serine proteases [65]. In vertebrates, serpins play crucial control in blood coagulation, fibrinolysis and inflammation. Dysfunction, deficiencies or over-expression of serpins can cause either abnormal bleeding or thrombosis [66]. The function of this protein in saliva of mosquitoes is related to host haemostasis regulation, seeming to act as a potent reversible inhibitor of the host factor Xa [67]. In *Ixodes ricinus* ticks, the molecule was also associated with inhibition of blood coagulation and fibrinolysis of the vertebrate host [68–70]. The consensus three-dimensional fold of serpins is comprised of a bundle of 8–9 α-helices and a β-sandwich composed of three β-sheets [71].

**Fig 6:**
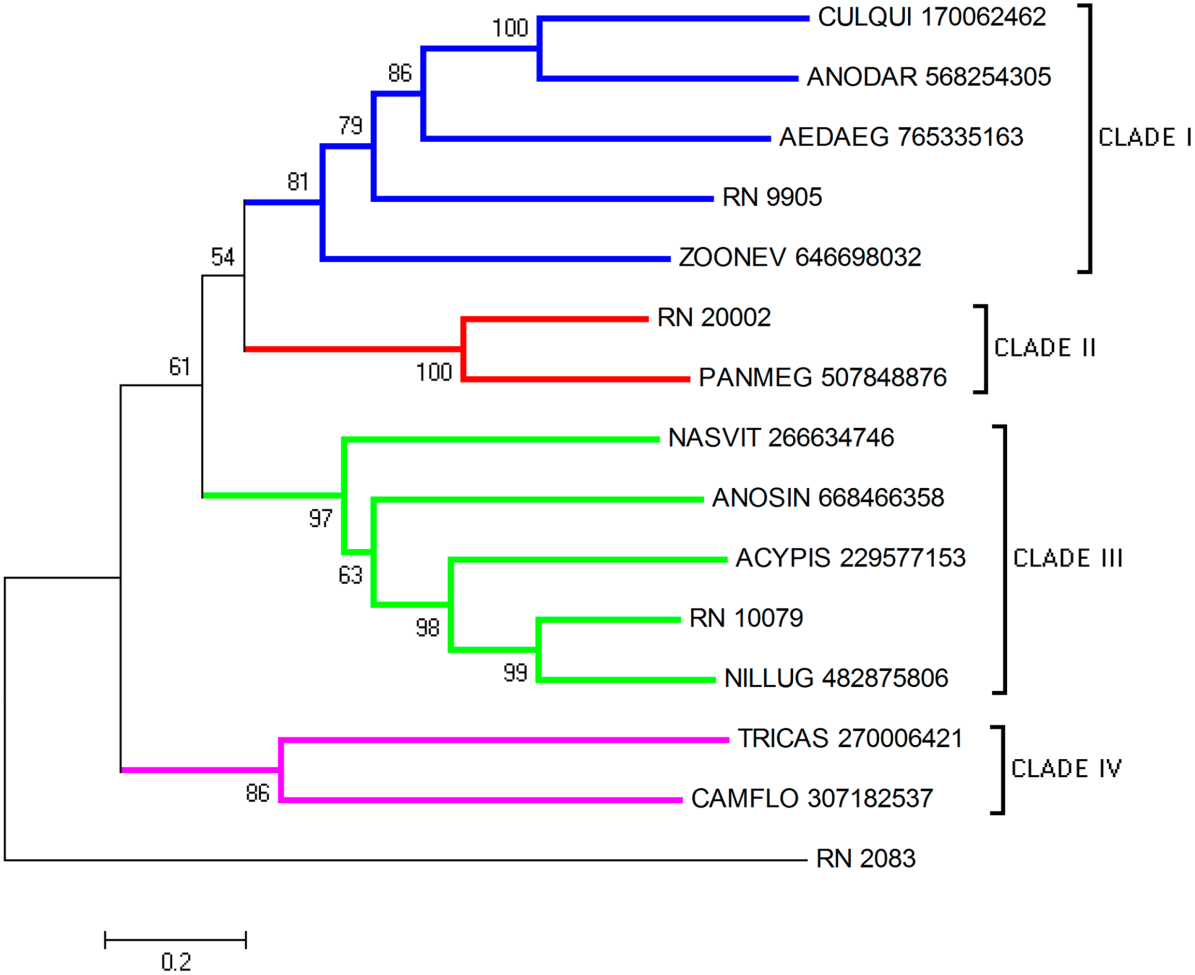
Phylogram of serpin proteins from *R*. *neglectus* SG transcriptome. Phylogenetic tree derived from the alignment of *R*. *neglectus* CDS and other insect sequences as described in Methods section. The bar represents 20% amino acid substitution.

Here, four CDS from *R*. *neglectus* sialotranscriptome were classified as serpins. The phylogram showed four clades with a good bootstrap support (Fig 6). Transcripts RN_9905, RN_20002 and RN_10079 grouped each one in a separate clade while the fourth CDS, RN_2083, seemed to be a distant divergent gene. In *R*. *neglectus* saliva this inhibitor might also function in the modulation of coagulation cascade.

### Proteases

#### Metalloprotease

One CDS found in the *R*. *neglectus* sialotranscriptome is related to the zinc-dependent metalloproteases from the astacin-like metalloproteases, a family of the metzincins superfamily. There are three conserved regions in proteins from this family. The first one is the distinguishing family signature sequence HEXXHXXGXXHE, which is the zinc-binding active site. The second region, RXDRD, is a hydrophilic region, and the third highly conserved region, MXY, is the methionine-containing turn (the Met-turn) [72–74].

**Fig 7:**
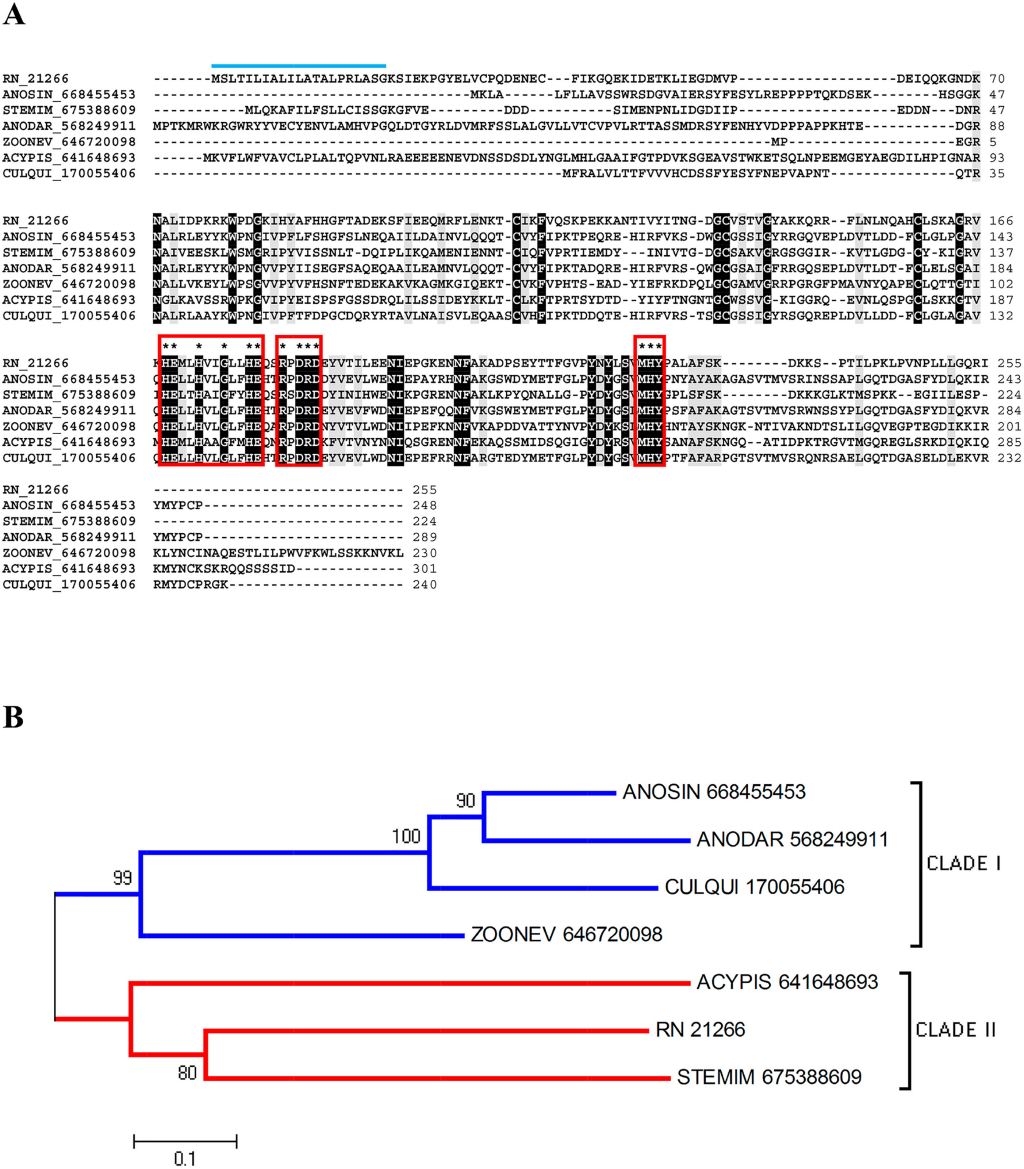
The secreted metalloprotease from *R*. *neglectus* SG transcriptome. (A) ClustalW alignment of the secreted metalloprotease from *R*. *neglectus* salivary transcriptome (RN_21266) and other metalloproteases sequences, identified as described in Methods section. The alignment indicates conserved residues in black and similar residues in gray background. The blue bar indicates the signal peptide indicative of secretion. The boxes limit the family signature sequences showing the determinant residues (black asterisk). (B) Phylogenetic tree derived from the alignment of *R*. *neglectus* CDS and other metalloproteases sequences as described in Methods section. The bar represents 10% amino acid substitution.

This gene family comprises many proteins from diverse species. In the venom of different spider species, there is a common toxin with the ability to hydrolyze fibrinogen and fibronectin [75–77], suggesting a relationship between this proteolytic activity with local hemorrhage, since fibronectin plays a role in platelet aggregation, blood vessel stability and wound healing [78]. Therefore, the biologic function of astacin-like proteases in triatomine saliva could be related to the maintenance of blood flow at the bite site. Astacin domain metalloproteases were already reported in *T*. *matogrossensis* sialotranscriptome [49]. The CDS RN_21266 is a full-length sequence containing a signal peptide indicative of secretion. Its alignment with other metalloproteases revealed the three conserved motifs of the family (Fig 7A). The phylogenetic tree suggests, with a good bootstrap support, that the secreted metalloproteases are closely related proteins (Fig 7B).

#### ADAMTS (ADAM with thrombospondin motifs)/Disintegrins

Two further members of the metzincin metalloprotease superfamily were identified in *R*. *neglectus* transcriptome and are related to the adamalysin/reprolysin family, which includes ADAM (A Disintegrin And Metalloproteinase domain) and ADAMTS (A Disintegrin And Metalloproteinase with Thrombospondin motifs). ADAMTS is a group of secreted, extracellular and multidomain proteases that have diverse roles in both mammals and invertebrates [79, 80]. They are cysteine-rich molecules that selectively block the function of integrin receptors on the cell membrane surface [81, 82], exhibiting a thrombospondin-like (TS) repeat and a cysteine-rich domain typical of disintegrins [79]. In this family, the third histidine in the family signature sequence containing three zinc ligands is followed by a conserved aspartic acid, HEXXHXXGXXHD. Moreover, it lacks the fifth zinc ligand and the methionine residue of the consensus Met-turn is placed within the sequence V/I-M-A/S [74, 79].

**Fig 8:**
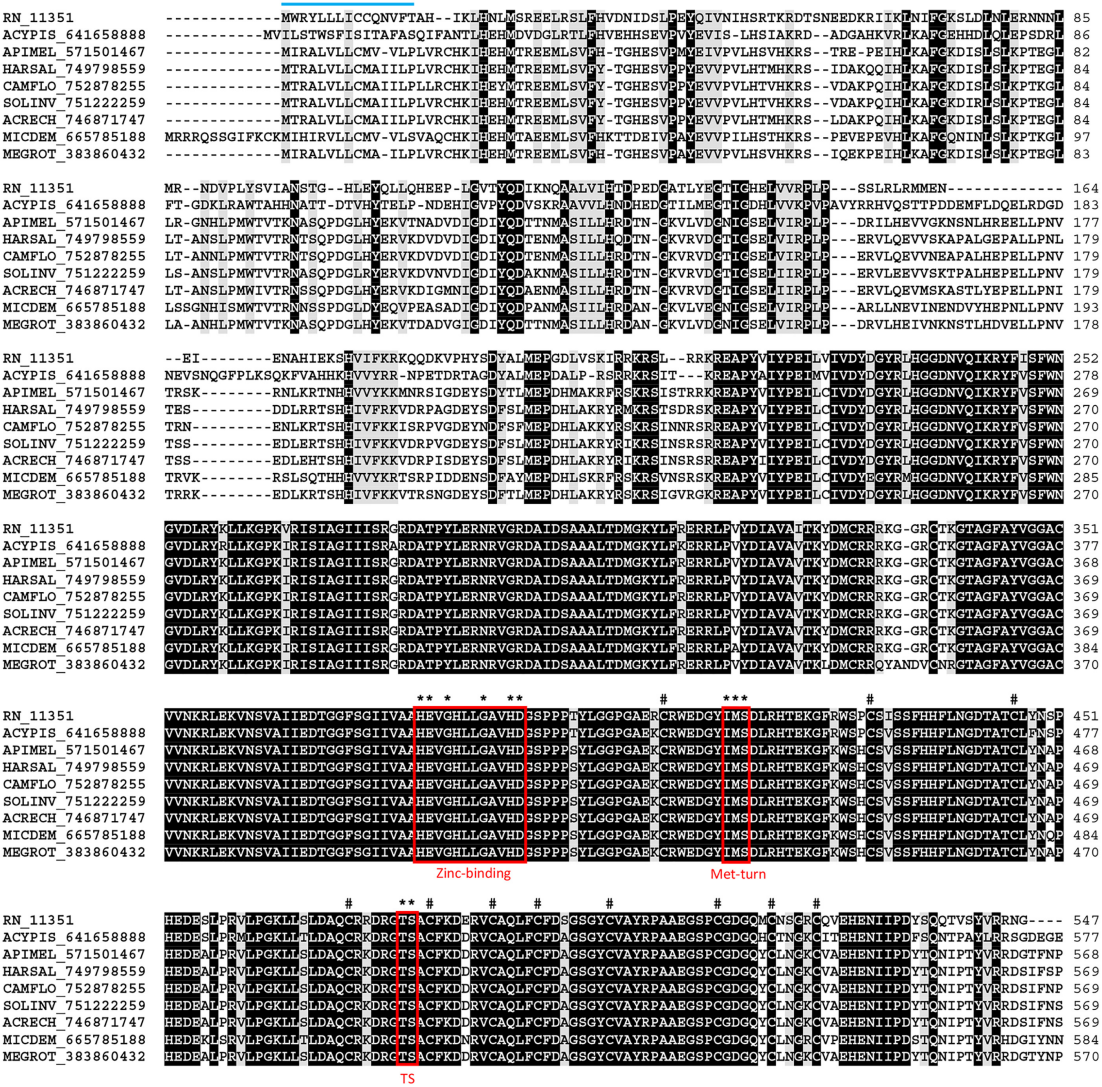
The ADAMTS sequence from *R*. *neglectus* SG transcriptome. ClustalW alignment of the ADAMTS sequence from *R*. *neglectus* salivary transcriptome (RN_11351) and other insect ADAMTS sequences, identified as described in Methods section. The alignment indicates conserved residues in black and similar residues in gray background. The bar indicates the signal peptide indicative of secretion. The boxes limit the family signature sequences showing the determinant residues (black asterisk). The symbols (black hash) above indicate the conserved cysteines.

Together with snake venom metalloproteinases (SVMP), ADAM and ADAMTS are a group of versatile molecules in viper venom that affects different elements in haemostasis [83]. The disintegrins can bind to platelets and act as potent inhibitors of platelet aggregation [84–86]. The molecule can also bind to endothelial cells [87, 88], as well as neutrophils and phagocytes [89]. Rhodostomin is a disintegrin that inhibits activity of LPS-treated monocytes via αvβ3 integrin affecting haemostasis, cell-cell interaction and suppresses tumor growth [90]. In hematophagous organisms, the disintegrins have been described in tick and leech saliva [91]. Here, one CDS related to disintegrins was identified in the *R*. *neglectu*s SG transcriptome. The alignment exhibited a high degree of homology between *R*. *neglectus* disintegrin sequence and others of the same family (Fig 8). The identification of ADAMTS is one of the main findings from the sialotranscriptome of *R*. *neglectus*.

#### Serine proteases and trypsin-like proteins

**Fig 9:**
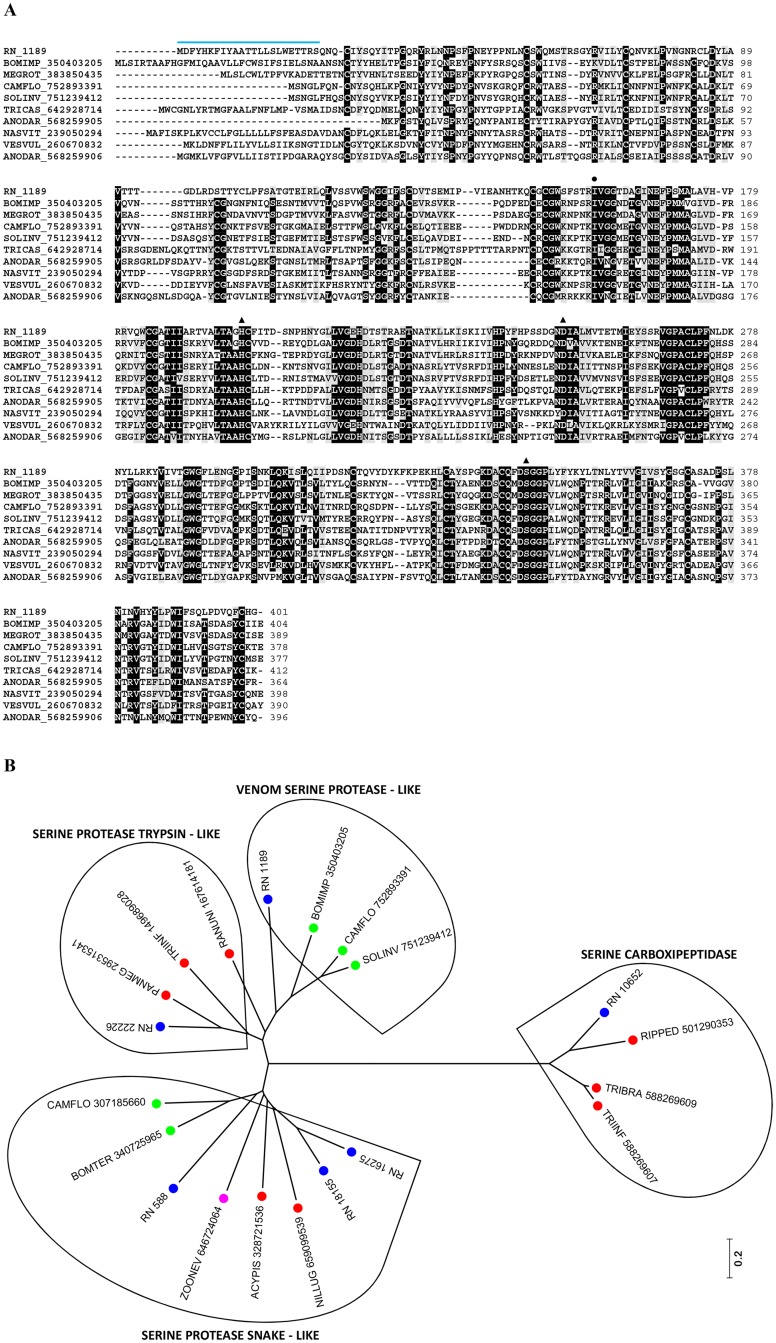
Serine proteases from *R*. *neglectus* SG transcriptome. (A) ClustalW alignment of a serine protease from *R*. *neglectus* SG transcriptome (RN_1189) and other serine proteases members, identified as described in Methods section. The alignment indicates conserved residues in black and similar residues in gray background. The blue bar indicates the signal peptide indicative of secretion. The symbols above the residues indicate (black circle) cleavage site and (black triangle) active site showing the HDS triad. (B) Phylogenetic tree was built from the alignment of *R*. *neglectus* CDS and other insect sequences as described in Methods section The bar at the bottom represents 20% amino acid substitution. The colored circles identify the sequences used: blue, *R*. *neglectus* sequences from SG transcriptome; red, Hemiptera order; green, Hymenoptera order; magenta, Dictyoptera order.

The *R*. *neglectus* SG transcriptome revealed serine proteases reads as the second most abundant group in the secreted class, comprising 820,619 reads. The majority of the sequences identified as serine proteases exhibited the trypsin domain (Tryp-SPc) of the CDD and Smart databases. RN_1189 was assembled from 768,048. Its alignment with serine proteases from other organisms revealed conserved residues located around the cleavage and active sites (Fig 9A), and the presence of a CUB (complement C1r/C1s, Uegf, Bmp1) domain, a structural motif of approximately 110 residues found almost exclusively in extracellular and plasma membrane-associated proteins. This domain is also present in honeybee allergens Api SI and Api SII, which are probably components of the honeybee defense system [92, 93].

Some serine proteases can function as regulators of coagulation. Thrombin can participate in this regulation by binding to thrombomodulin, a membrane protein present in host endothelial cells. This complex is able to activate Protein C (a serine protease), which acts as a potent anticoagulant enzyme by inactivating factors V and VIII, impairing thrombus progression [94, 95]. In snake venom, blockage of thrombus formation by serine proteases has also been reported. SPSV (Serine Protease Snake Venom) releases a unique fibrinopeptide that produces only instable monomers of fibrin, leading to clots that are rapidly dispersed [96]. Although the specific role in hematophagous saliva is still unknown, an active serine protease was described in *T*. *infestans* [97], as well as in horse fly *Tabanus yao* saliva, which functions as a fibrinogenolytic enzyme [98]. RN_22226, RN_21634, RN_19989, RN_17969, and RN_10652 were matched by blastp to serine proteases of *T*. *infestans*, *T*. *braziliensis*, *Panstrongylus megistus*, and *R*. *prolixus*. Serine proteases also play important roles in fertilization, embryonic development, and in the processes of molting and metamorphosis of insects [99, 100]. In our sample, the triatomines did not show any sign of larval molting at SG dissection.

Sequences containing CLIP, LDLa and SUSHI domains, which are cysteine-stabilized structures for molecular recognition, were also identified. The CLIP domain is restricted to the Arthropoda and was found N-terminally to the Tryp-SPc domain of RN_16275, RN_18155, and RN_7118. Both domains belong to the serine proteases of the trypsin-like S1 family, that are typically secreted enzymes associated with extracellular proteolysis [101]. CLIP domain has been suggested to be important for dimerization, mediating specific protein-protein interactions involved in the regulation of serine protease activities. The LDLa domain was identified in RN_12992, RN_12776, RN_12432, and RN_21634. The last two sequences also presented the SUSHI motif of smart database which is known as CCP (Complement Control Protein) module, containing approximately 60 amino acid residues identified in several proteins of the complement system. These *R*. *neglectus* putative secreted serine proteases may play critical roles in many key biological processes as blood coagulation and immunity. In the vertebrate hosts, allergenicity may reinforce the toxic effect of serine proteases, independently of their catalytic activity, as proposed by Georgieva and colleagues [93].

The phylogram clearly showed four different groups (Fig 9B), indicating the expression of at least four genes related to serine proteases in the saliva of *R*. *neglectus*. In regard to the large amount of reads, the results observed here suggests that, to *R*. *neglectus*, the serine proteases arise as an important salivary secreted gene family, a probably evolutionary adaptation where the protein could gain a new function as a result of selective pressure for the blood-feeding behavior success. These proteases could act in the vertebrate host, as well as in the insect, on pathogens that were ingested with blood. Further experiments are necessary to address the possible roles of those genes on the biology of *R*. *neglectus*.

### OBP

**Fig 10:**
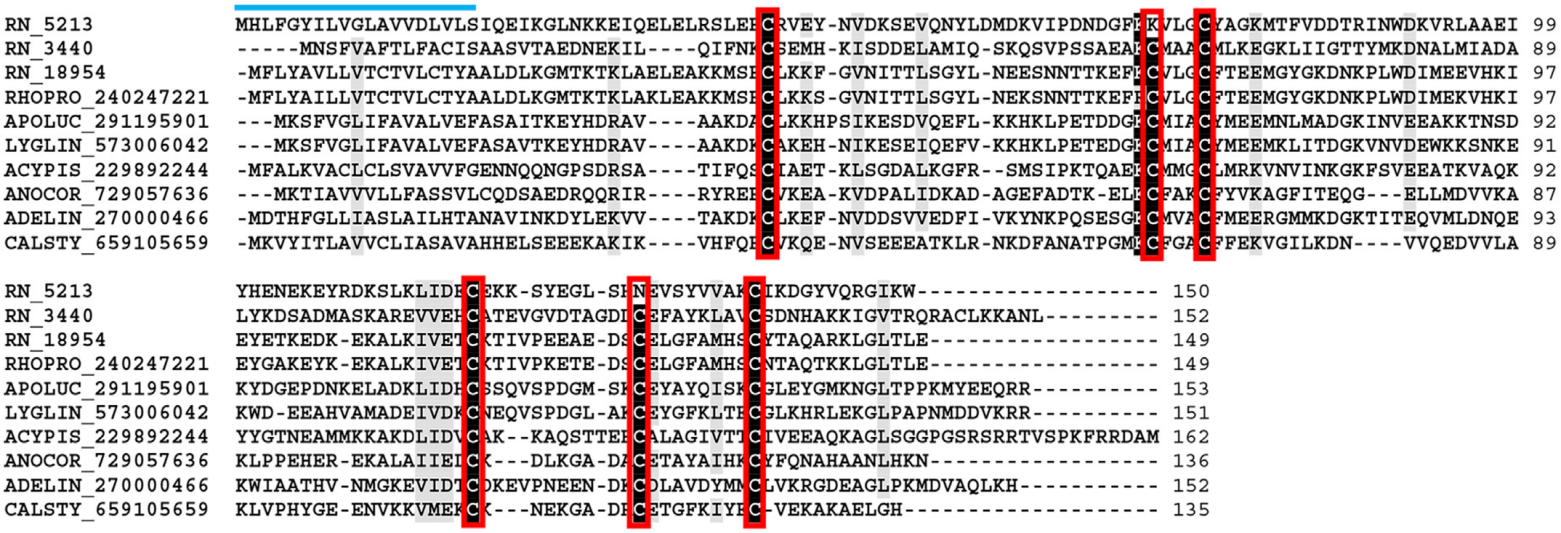
The secreted OBP family from *R*. *neglectus* SG transcriptome. ClustalW alignment of secreted OBPs from *R*. *neglectus* SG transcriptome (RN_5213, RN_3440 and RN_18954) and other members from the OBP family, identified as described in Methods section. The alignment indicates conserved residues in black and similar residues in gray background. The blue bar indicates the signal peptide indicative of secretion and the boxes, the six conserved cysteines.

The odorant-binding protein (OBP) family is a chemosensory protein ubiquitous in insects commonly associated with solubilizers and carriers of odorants and pheromones. Although associated with chemosensory organs, in recent times this family has been related to other roles such as hydrophobic chemical transportation [102]. The OBPs are characterized by a variable amino acid sequence, but conserve a pattern of six conserved cysteines residues paired to form three disulfide bridges [103]. The folding is a typical six α-helices assembled in a compact and stable structure [104, 105]. Eleven CDS containing protein sequences related to OBPs were recognized in our transcriptome analysis, all possessing signal peptide prediction. The conserved cysteine residues of *R*. *neglectus* CDS (Fig 10) were seen during alignment. Phylogenetic analysis with good bootstrap support shows Clade I containing most *R*. *neglectus* sequences grouped with *R*. *prolixus* OBP. However, RN_3440 was grouped in Clade III, suggesting this is a more distant OBP (S4 Fig).

### The Proteome of *R*. *neglectus* Saliva

*R*. *neglectus* saliva content was tryptic digested and subjected to mass spectrometry to validate the analysis of the transcripts possibly associated with secreted products. Among the 73 identified secreted proteins groups, 48 were from the lipocalin family, including triabin, pallidipin and nitrophorin proteins, reaffirming their abundance (Table 4). Other soluble proteins, predicted as being secreted by these arthropods, were: secreted metalloprotease, antigen-5, serpin and trypsin-like protease, each with at least one observation. It is intriguing that only one serine protease was detected by proteomic analysis, regardless the high number of transcripts reads assigned to this subclass of putative secreted proteins (Table 3). There are several possible explanations for this observation. First, the proteins are expressed in a such a small amount not detectable by our proteomic approach; second, the proteins are not secreted; third and most likely, these proteins present in SGs are expressed upon specific physiological conditions, such as during stimulation of salivation (feeding). In addition, it is also possible that those enzymes have both intracellular and extracellular functions as many other proteases do.

**Table 4:** Classification and abundance of proteins from the salivary proteome of *R*. *neglectus* based on LC-MS/MS.

Class	No. of protein groups	% Total
Lipocalin–Triabin	34	46.58
Lipocalin–Nitrophorin	17	23.29
Others	13	17.81
Conserved secreted protein	2	2.74
Antigen-5/SCP	1	1.37
Inositol polyphosphate phosphatase	1	1.37
Metalloprotease	1	1.37
Protease inhibitor–Serpin	1	1.37
Chitinase-like lectin	1	1.37
Trypsin-like protease	1	1.37
Juvenile hormone related	1	1.37
**Total**	**73**	**100**

### Comparison of Protein Contents between *R*. *neglectus* and *R*. *prolixus*

A comparative blastp analysis was employed to address the similarity of the SGs proteins from *R*. *neglectus* compared to *R*. *prolixus*. The two species do not show high evolutionary divergence, presenting at least 80% identity in analyzed sequences (Table 5), suggesting both species share a common ancestral lineage. As described before, this high degree of protein similarity was also seen with *R*. *brethesi* and *R*. *robustus* in the Amazon rainforest [106].

**Table 5:** Identities of *R*. *neglectus* proteins compared to *R*. *prolixus* (v 3.0) proteins by blastp.

Class	Average identity	SE*	N
Secreted	86.46	1.47	200
Immunity	91.17	3.76	35
Transporters and channels	92.33	1.22	114
Extracelular matrix	94.07	1.81	43
Transposable elements	94.64	2.50	22
Protein export	95.09	0.84	166
Signal transduction	95.39	0.54	285
Storage	95.50	1.44	8
Cytoskeletal	95.64	0.96	88
Unknown conserved	95.99	0.44	369
Protein modification	95.99	0.78	144
Metabolism	96.28	0.45	405
Transcription machinery	96.50	0.50	250
Proteasome machinery	96.59	0.80	122
Nuclear regulation	96.65	0.75	95
Nuclear export	96.67	1.05	12
Unknown	97.04	1.47	24
Detoxification	97.15	1.16	48
Transcription factor	97.43	0.63	53
Protein synthesis	97.95	0.42	180
**Total**			**2,663**

*SE: standard error

Identity among sequences was greater in housekeeping class members, showing that these proteins have a lower evolutionary rate than those of the secreted class. This indicates that antihaemostatic proteins evolve faster after divergence. Different molecular mechanisms may be responsible for the variation between these closely related *Rhodnius* species, expanding their biological diversity patterns. The particularity of each species could be related to their different habitats, including different prey and abiotic factors.

### Final Considerations

Hematophagy evolved independently at least six times in approximately 15,000 species allowing for adaptation to an existing complex host haemostatic system [5, 107]. Thus, many salivary molecules target different pathways for the insect to achieve a successful blood meal. Here, we described *R*. *neglectus* sialome in all its complexity to expand our knowledge of the salivary proteins from hematophagous triatomine bugs.

*R*. *neglectus* is considered of secondary importance in the transmission of *T*. *cruzi*, causative agent of Chagas disease. The analysis of salivary secretory products of *R*. *neglectus* that might be involved in vector-host interactions share similarity with other triatomine species, which can also be infected by and transmit the protozoan.

It is possible that the expression of putative trypsin-like serine proteases in the SGs of *R*. *neglectus* correlates with blood sources of this species of triatomine. Their role and that of other secreted class, hypothetical and conserved secreted proteins, in hematophagy should be analyzed in future works, and we accentuate that sialome study is still an open field for new discoveries.

## Supporting Information

S1 Fig Antigen-5 proteins from *R*. *neglectus* SG transcriptome. ClustalW alignment of antigen-5 members from *R*. *neglectus* salivary transcriptome (RN_21331, RN_21434 and RN_21812) and other hemiptera sequences, identified as described in Methods section. The alignment indicates conserved domains in black and similar domains in gray background.
(TIF)

S2 Fig The dipetalogastin from *R*. *neglectus* SG transcriptome. ClustalW alignment of a dipetalogastin member from *R*. *neglectus* salivary transcriptome (RN_1079) and other sequences from dipetalogastin family, identified as described in Methods section. The alignment indicates conserved residues in black and similar residues in gray background. The boxes indicate conserved motifs, and the blue bar indicates the signal peptide indicative of secretion.
(TIF)

S3 Fig Pacifastin members from *R*. *neglectus* SG transcriptome. ClustalW alignment of pacifastin members from *R*. *neglectus* salivary transcriptome (RN_17301 and RN_20047) and other sequences from the pacifastin family of proteins, identified as described in Methods section. The alignment indicates conserved residues in black and similar residues in gray background. The bars indicate the four conserved pacifastin motifs.
(TIF)

S4 Fig Phylogram of the secreted OBP family from *R*. *neglectus* SG transcriptome. Phylogenetic tree was built from the alignment of *R*. *neglectus* CDS and other OBP sequences as described in Methods section. The bar represents 20% amino acid substitution.
(TIF)
