# Supplementary figures and images for: A Deep Insight into the Sialome of Rhodnius neglectus, a Vector of Chagas Disease
Source: PLoS Negl Trop Dis. 2016 Apr 29;10(4):e0004581. doi: 10.1371/journal.pntd.0004581 (PMC4851354; doi:10.1371/journal.pntd.0004581)

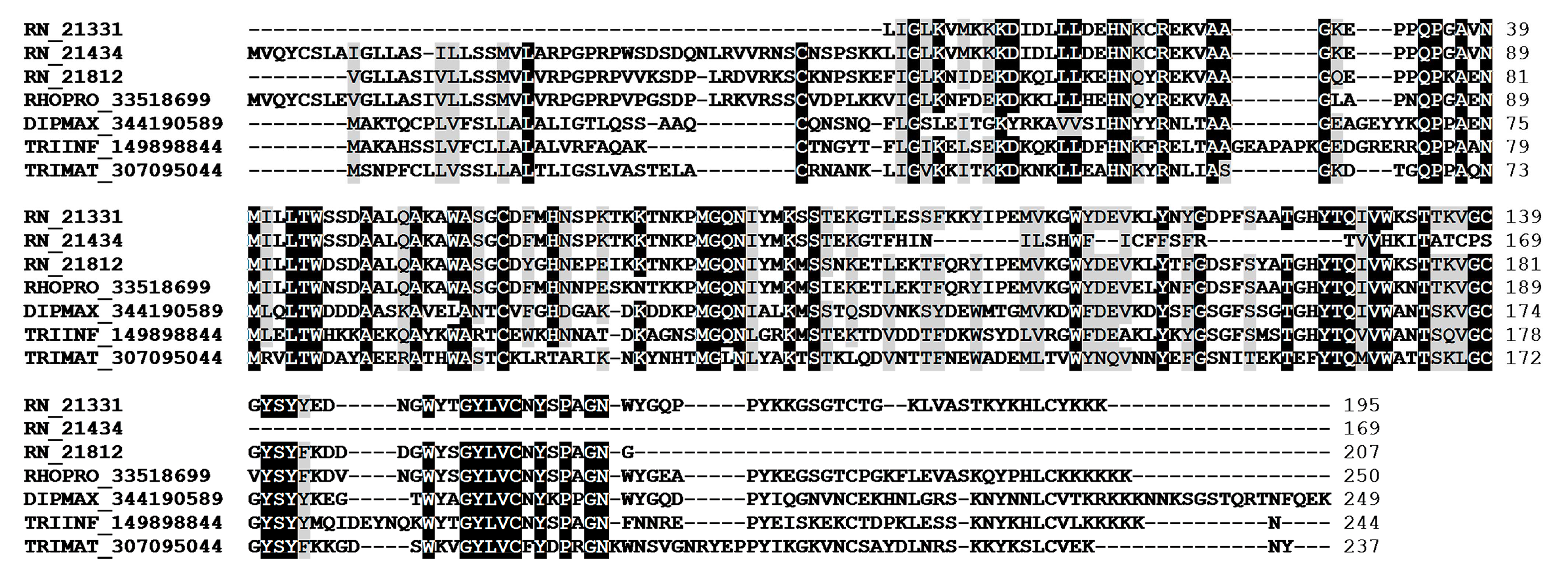

Supplement: S1 Fig — ClustalW alignment of antigen-5 members from R. neglectus salivary transcriptome (RN_21331, RN_21434 and RN_21812) and other hemiptera sequences, identified as described in Methods section. The alignment indicates conserved domains in black and similar domains in gray background. (TIF) [file pntd.0004581.s001.tif]

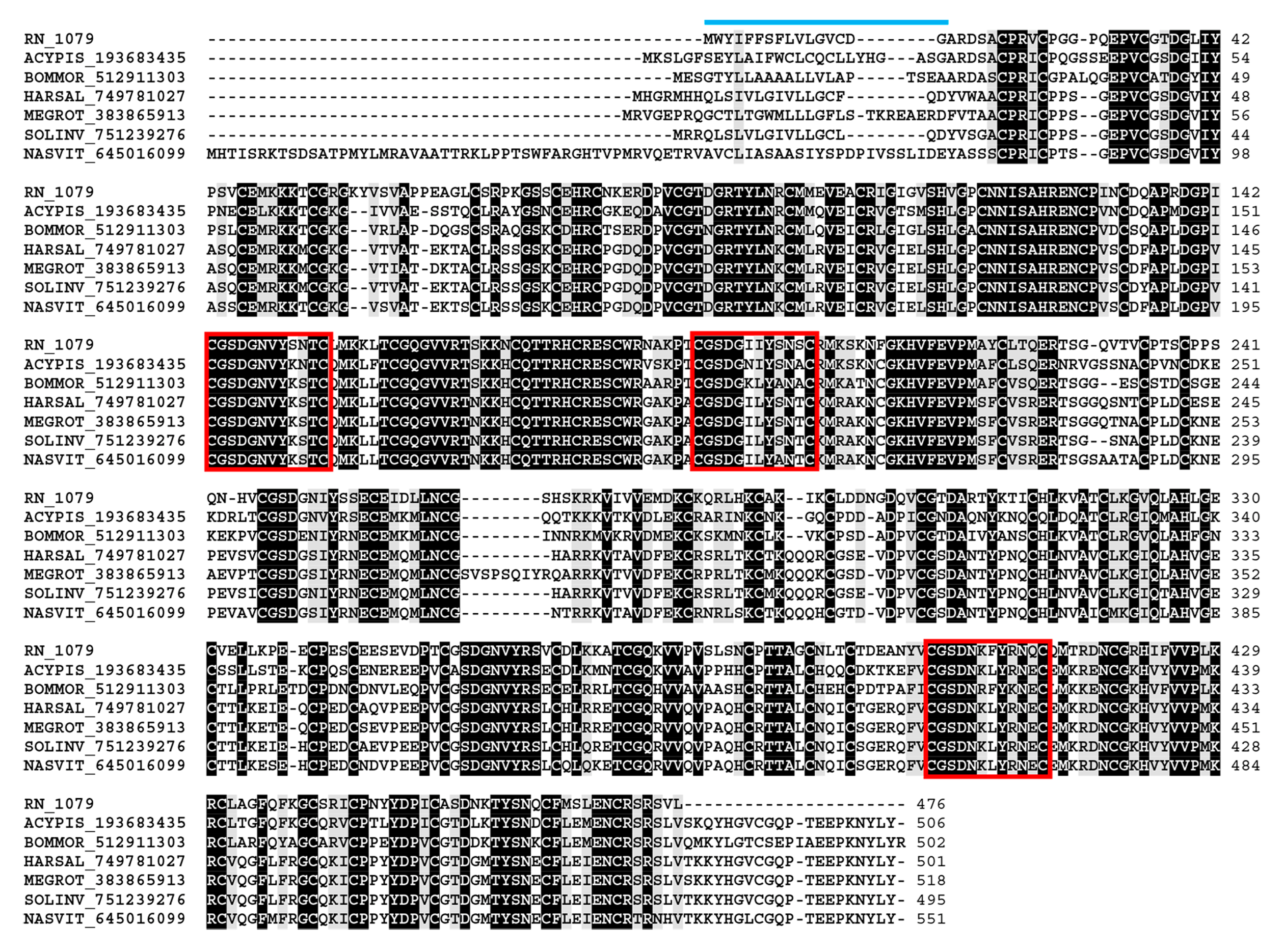

Supplement: S2 Fig — ClustalW alignment of a dipetalogastin member from R. neglectus salivary transcriptome (RN_1079) and other sequences from dipetalogastin family, identified as described in Methods section. The alignment indicates conserved residues in black and similar residues in gray background. The boxes indicate conserved motifs, and the blue bar indicates the signal peptide indicative of secretion. (TIF) [file pntd.0004581.s002.tif]

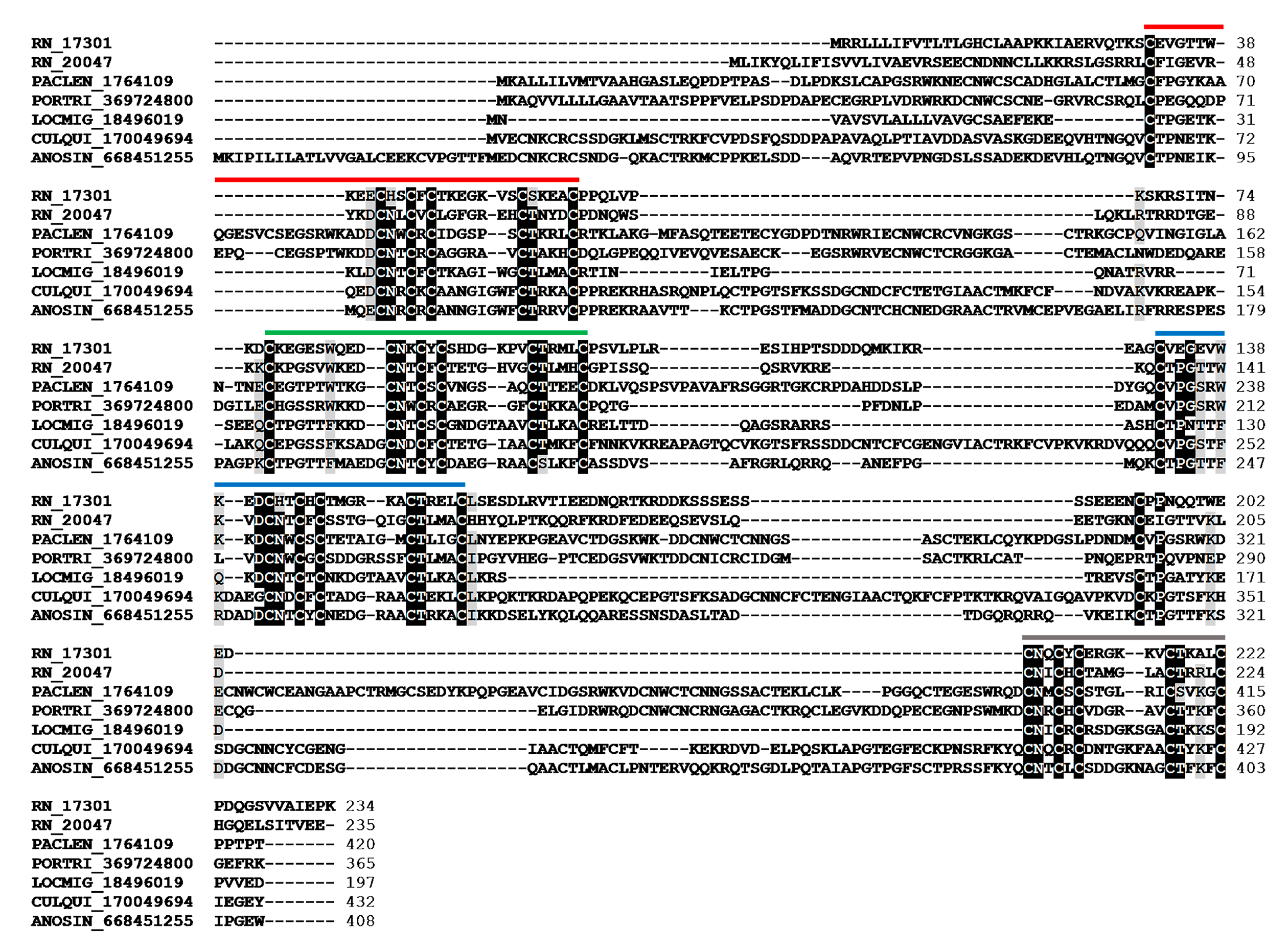

Supplement: S3 Fig — ClustalW alignment of pacifastin members from R. neglectus salivary transcriptome (RN_17301 and RN_20047) and other sequences from the pacifastin family of proteins, identified as described in Methods section. The alignment indicates conserved residues in black and similar residues in gray background. The bars indicate the four conserved pacifastin motifs. (TIF) [file pntd.0004581.s003.tif]

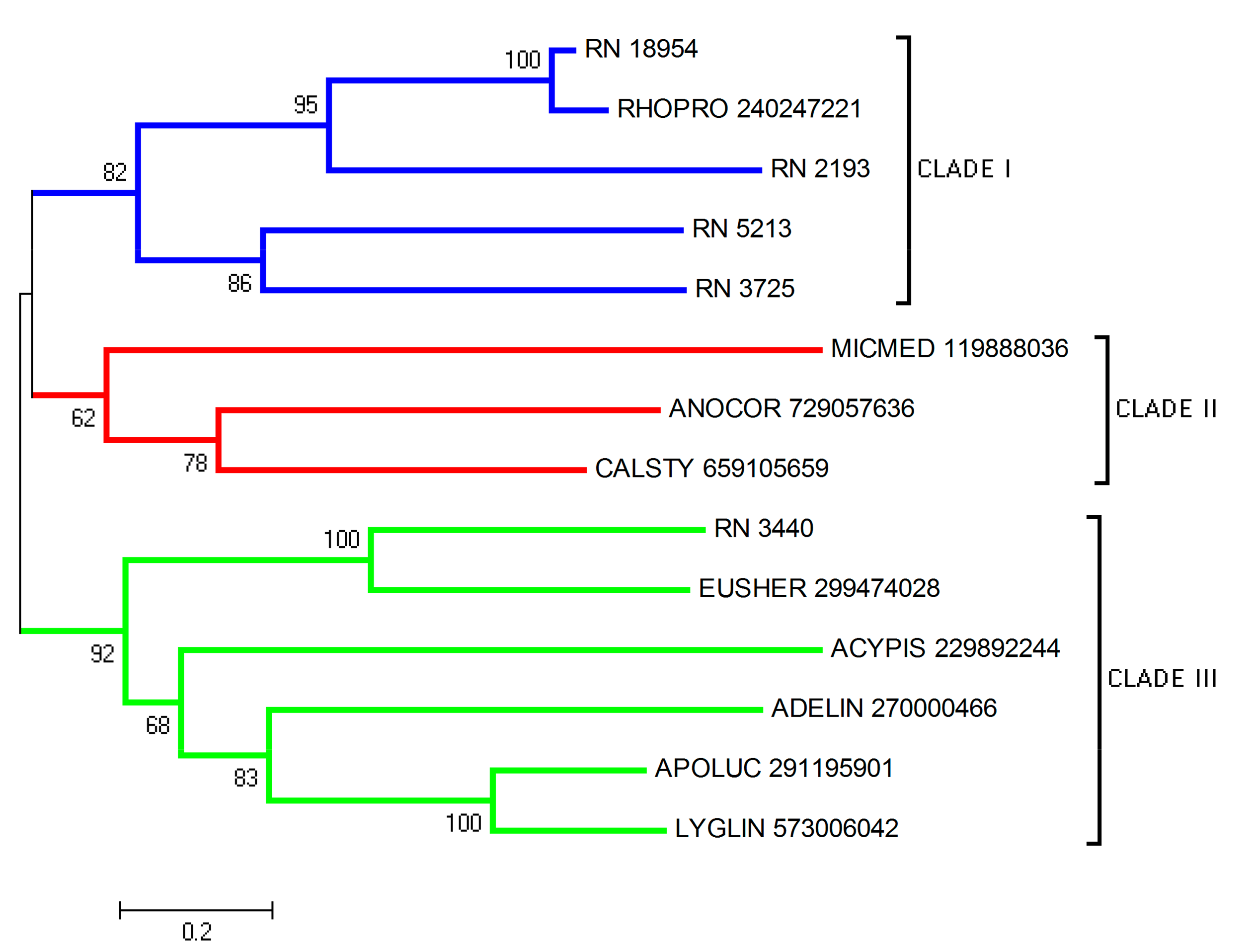

Supplement: S4 Fig — Phylogenetic tree was built from the alignment of R. neglectus CDS and other OBP sequences as described in Methods section. The bar represents 20% amino acid substitution. (TIF) [file pntd.0004581.s004.tif]
